# Supplementary material for: Polymorphisms in Pfkelch13 domains before and after the introduction of artemisinin-based combination therapy in Southwest Nigeria
Source: PLoS One. 2025 Mar 31;20(3):e0316479. doi: 10.1371/journal.pone.0316479 (PMC11957316; doi:10.1371/journal.pone.0316479)
Supplement: Supporting information 2 — (ZIP) [file pone.0316479.s002.zip › 057KN2R_PREMIX_Plate_KELCH2_F11.pdf]

Samples: 12305
Bases: 1036
Average spacing: 12

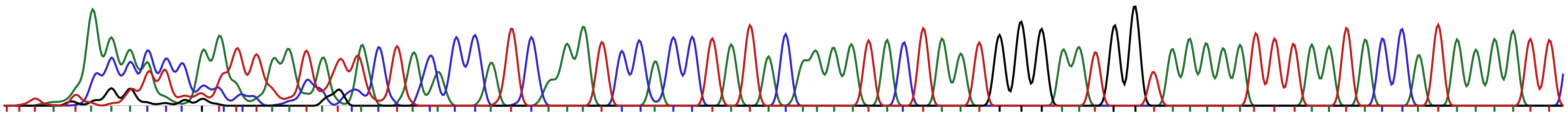


T T T A T A A A C C C G T C T C T A A T A T C A C T A C A C C C A T C A A A T C C A C C T A T A C A A A A T A C T A A T G G G A A T G G T A A A A T T T A A T A C C A T A A A A T T

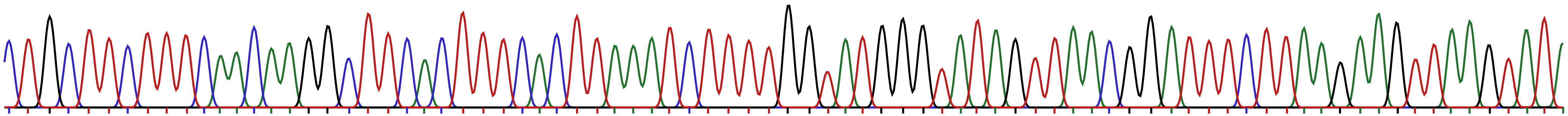


C T G C T T C T T T C A A C A A G G C T T C A C T T T C A C T T A A A T C T T T T G G T A T G G G T A T A G T T A A C G G A T T T C T T A A G A A G T T A A G T A T A

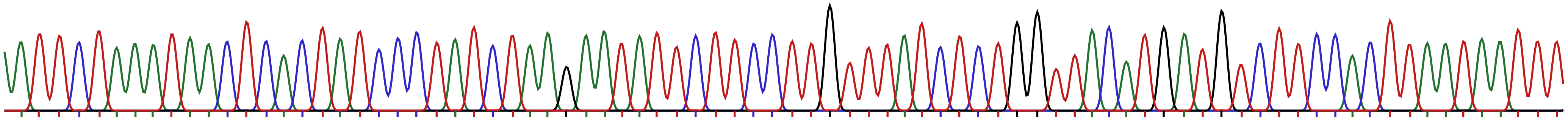


A T T C T A A T A A C T C A C T A T C C C T A T C T A A G A A T A T T C T T C C T T G T T T A T C T C T G G T T A C A T G A T G T C T T C C A C T T A A T A A T T T

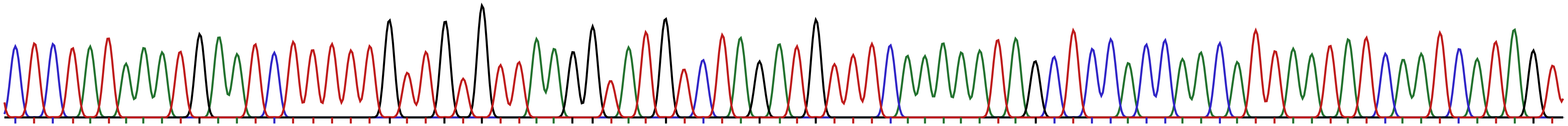


C T C T A T A A A T G A A T C T T T T T G T T G T G T T A A G G T A T G T C T A G A T G T T T C A A A A A T A G C T C C A C C A A C A T T A A T A T C A A T C A T A G T

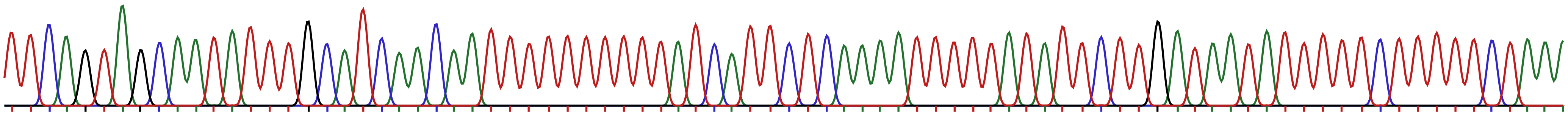


T T C A G T A G C A A T A T T T G C A T C A A C A A T T T T T T T T T A T C A T T C T C A A A A T T T T T A T A T T C T T G A T A A T A T T T T T C T T T T T C T A A A

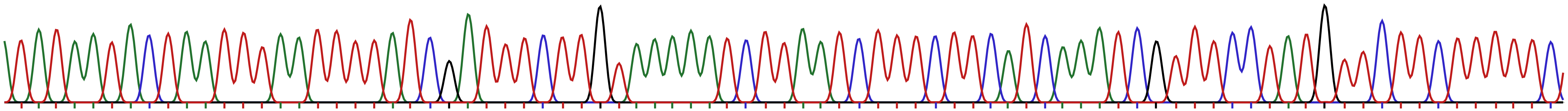


T A T A A T A C T A A T T T A A T T T T A T C G A T T T C T T G T A A A A A T C T T A A T C T T T C T T C A T C A A A T C G T T T C C T A T G T T C T T C T T T T T C

Samples: 12305
Bases: 1036
Average spacing: 12

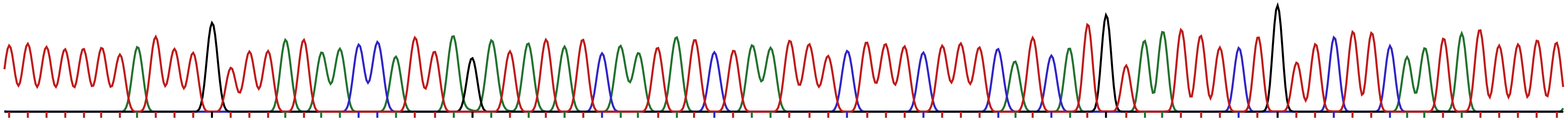

510 520 530 540 550 560 570 580 590
T T T T T T T A T T T G T T T A T A A C C A T T A G A T A T A T C A A T A T C T A A T T T C T T T C T T T C A T C A T G T A A T T T C T G T T C T T C A A T A T T T T T

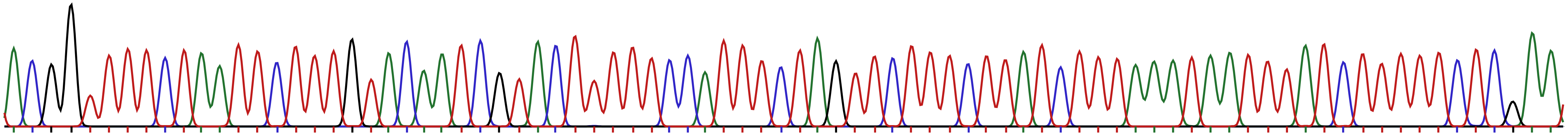

600 610 620 630 640 650 660 670
A C G G T T T T C T A A T T C T T T G T A C A A T C G T A C T T T T T C C A T T T C T A G T T C T T T C T T A T C T T T A A A T A A T T T A T C T T T T T C T C G A A

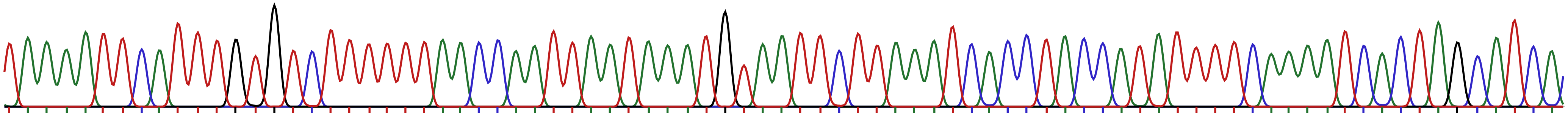

680 690 700 710 720 730 740 750
T A A A A T T C A T T T G T G T C T T T T T T A A C C A A T T A A T A A A T G T A A T T C T T A A A T C A C C T A C C A T A T T T T C A A A A T C A C T A G C A T C A

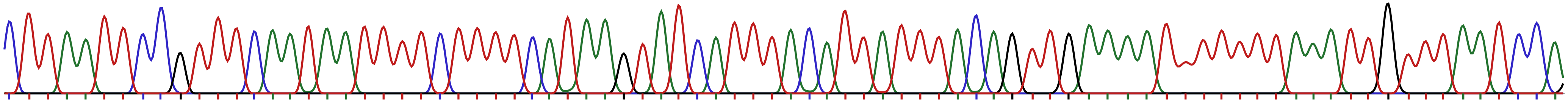

760 770 780 790 800 810 820 830 840
C T T A A T T C C G T T T C A A T A A T T T T C T T T T C A T A A G T A T C A T T T A C A T T A T T T A C A G T T G A A A A T T T T T T T A A A T T G T T T A A T C C A

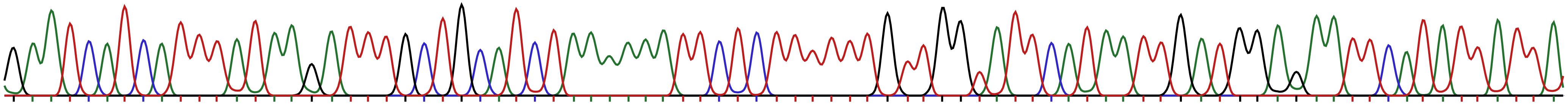

850 860 870 880 890 900 910 920
G A A T C A T C A T T T A T A A G A T T T G C T G C A T C T A A A A A A T T C T C T T T T T T G T T G G T A T T C A T A A T T G A T G G A G A A T T C A T A T T A T T A

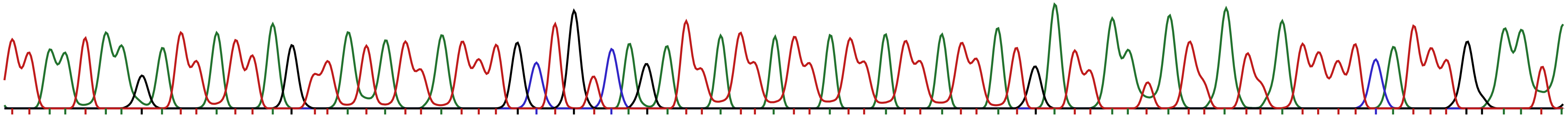

930 940 950 960 970 980 990 1000 1010
T T A A T A A G A T T A T T A G TT A T A T T A T T T G C T G T C A G A T T A T T A T T A T T A T T A T T A T G A T T A A T A T T A T T A T T T T C A T T T GG A A T A

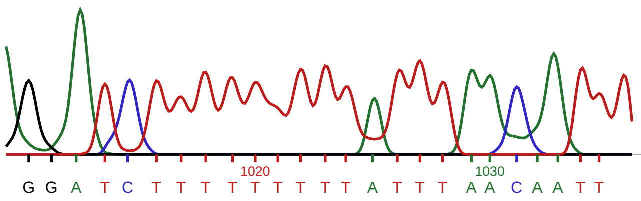
1020
1030
G G A T C T T T T T T T T T A T T T A A C A A T T
